# Local Electric Field-Incorporated In-Situ Copper Ions Eliminating Pathogens and Antibiotic Resistance Genes in Drinking Water

**DOI:** 10.3390/antibiotics13121161

**Published:** 2024-12-02

**Authors:** Ruiqing Li, Haojie Dai, Wei Wang, Rulin Peng, Shenbo Yu, Xueying Zhang, Zheng-Yang Huo, Qingbin Yuan, Yi Luo

**Affiliations:** 1 School of Environmental Science and Engineering, Nanjing Tech University, Nanjing 211816, China; 18705629251@163.com (R.L.); wangwei@njtech.edu.cn (W.W.); pengrulin@njtech.edu.cn (R.P.); zhangxueying@njtech.edu.cn (X.Z.); 2 State Key Laboratory of Pollution Control and Resource Reuse, School of the Environment, Nanjing University, Nanjing 210023, China; 2020201491@ruc.edu.cn; 3 School of Environment and Natural Resources, Renmin University of China, Beijing 100872, China; yushenbo@nju.edu.cn (S.Y.); luoy@nju.edu.cn (Y.L.); 4 School of Chemistry and Life Resources, Renmin University of China, Beijing 100872, China

**Keywords:** electroporation, pathogens, antibiotic resistance genes, copper ions

## Abstract

Background/Objectives: Pathogen inactivation and harmful gene destruction from water just before drinking is the last line of defense to protect people from waterborne diseases. However, commonly used disinfection methods, such as chlorination, ultraviolet irradiation, and membrane filtration, experience several challenges such as continuous chemical dosing, the spread of antibiotic resistance genes (ARGs), and intensive energy consumption. Methods: Here, we perform a simultaneous elimination of pathogens and ARGs in drinking water using local electric fields and in-situ generated trace copper ions (LEF-Cu) without external chemical dosing. A 100-μm thin copper wire placed in the center of a household water pipe can generate local electric fields and trace copper ions near its surface after an external low voltage is applied. Results: The local electric field rapidly damages the outer structure of microorganisms through electroporation, and the trace copper ions can effectively permeate the electroporated microorganisms, successfully damaging their nucleic acids. The LEF-Cu disinfection system achieved complete inactivation (>6 log removal) of *Escherichia coli* O157:H7, *Pseudomonas aeruginosa* PAO1, and bacteriophage MS2 in drinking water at 2 V for 2 min, with low energy consumption (10^−2^ kWh/m^3^). Meanwhile, the system effectively damages both intracellular (0.54~0.64 log) and extracellular (0.5~1.09 log) ARGs and blocks horizontal gene transfer. Conclusions: LEF-Cu disinfection holds promise for preventing horizontal gene transfer and providing safe drinking water for household applications.

## 1. Introduction

**Figure 1:**
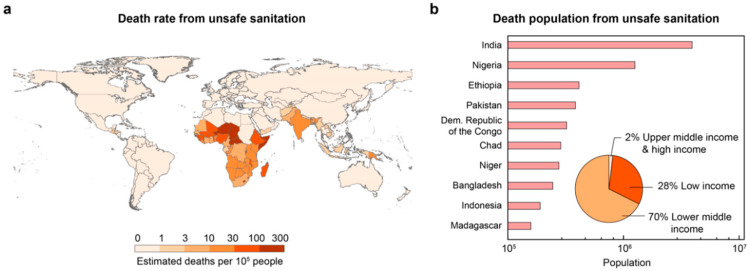
Deaths from unsafe sanitation. (**a**) Estimated annual number of deaths due to unsafe sanitation per 100,000 people. (**b**) The total death population from unsafe sanitation. Data source: Global Burden of Disease (2024).

Waterborne pathogen infection has become one of the most serious threats to public health. According to the latest estimates from the World Health Organization, more than 1.4 million people die each year due to inadequate sanitation, with 98% of deaths occurring in low and lower–middle–income countries, such as the Sub-Saharan, Southeast Asia, South Asia, and Central America (Figure 1). In disaster areas and isolated communities where municipal facilities are not readily available, chemical-free, energy-efficient disinfection methods for inactivating pathogens in drinking water are essential to prevent epidemic outbreaks. In addition, bacteria carrying ARGs are widely present in drinking water [1]. These harmful genes can remain active in the environment and have the potential to be transferred to pathogens from the One Health perspective, leading to infections that are difficult to treat with conventional antibiotics [2,3]. Therefore, it is essential to develop a highly efficient, cost-effective disinfection method for the simultaneous removal of pathogens and ARGs just before drinking, without relying on intensive energy input and chemical dosage.

However, the most commonly used drinking water disinfection technology, chlorination, has several drawbacks which include, continuous chemical dosing, the formation of harmful DBPs, and the potential spread of ARGs [4,5]. Alternative methods, such as ultraviolet (UV) disinfection and membrane filtration, are limited by intensive energy consumption and high construction and operating costs [6,7,8]. In addition, current disinfection methods are ineffective at targeting and damaging harmful genes such as ARGs and have difficulties preventing the spread of risk genes (i.e., blocking horizontal gene transfer) [9,10,11,12]. Thus, if a disinfection method that can simultaneously remove pathogens and harmful genes in drinking water requires a low energy input without external chemical dosing for household applications (e.g., water pipes), it can undoubtedly protect people from pathogen infection. However, the current water disinfection methods do not meet these critical needs.

Electroporation, a physics-based disinfection method that relies on a strong electric field to damage the outer structure of microorganisms (bacterial membrane and viral capsid), holds great promise for enabling chemical-free and point-of-use disinfection [13,14,15]. However, the required strong electric field (>10^6^ V/m) poses a major challenge for practical application, as a high external voltage (at least several hundred V) is usually required to generate a sufficient field [16,17]. Researchers have attempted to use high aspect ratio nanomaterials (such as nanowires, nanotubes, and nanorods) to generate a strong local electric field based on the lightning rod effect at low applied voltage [18,19,20]. However, the unavoidable release of nanomaterials limits their application for providing safe water just before drinking. In addition, the fabrication of nanomaterial-assisted electrodes for electroporation requires toxic/dangerous reagents (e.g., phosphine for fabricating cuprous phosphide nanowires) and high energy consumption (heating or hydrothermal process).

When using a thin metal wire with a diameter of less than 100 μm, a strong local electric field (>10^4^ V/m) can be generated near the wire surface with an external voltage of several V [21]. This is because free charges accumulate in the region with a small radius of curvature, making the thin conductive wire ideal for charge accumulation to generate an enhanced local electric field [22]. While this enhanced local electric field is not enough for immediate bacterial or viral inactivation, it can affect the microbial outer structure through reversible electroporation, potentially integrating with another antimicrobial process [23,24,25]. Researchers found that using a copper wire as an anode can generate trace copper ions (Cu^2+^) near the electrode surface with a low external voltage (1–3 V) [26]. This in-situ released Cu^2+^ can effectively transfer into the electroporated bacteria and cause rapid disinfection [26,27]. However, only model bacteria such as *Escherichia coli* (*E. coli*) have been tested, and the feasibility of inactivating pathogens and viruses in practical drinking water is questionable. In addition, the effectiveness of damaging ARGs to block horizontal transfer to eliminate risk gene transfer is still unknown.

In this study, we introduce an LEF-Cu disinfection method for the simultaneous inactivation of pathogens and destruction of ARGs in drinking water. A copper wire anode (100 μm diameter), which can be easily placed in the middle of a household water pipe, can generate LEF and Cu^2+^ to achieve a synergistic effect for pathogen disinfection and ARG removal without external chemical dosing. The trace copper ions can effectively transfer into the electroporated microorganisms caused by the local electric field to damage basic groups of genes through complex reactions. The LEF-Cu method achieved complete disinfection of pathogens and viruses and effectively damaged ARGs in drinking water. Our work provides a proof-of-concept for a chemical-free, energy-efficient method that can be readily applied to point-of-use applications as a last line of defense to protect people from pathogen infection.

## 2. Results and Discussion

### 2.1. Disinfection Performance and Mechanisms of LEF-Cu Method

**Figure 2:**
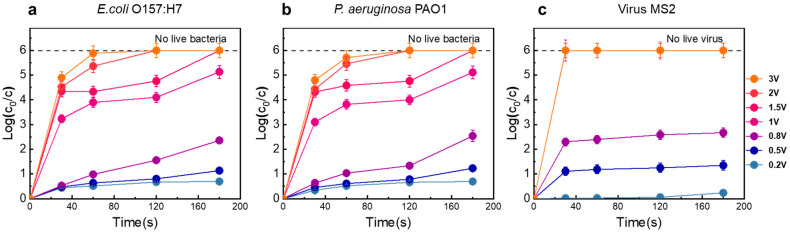
Disinfection performance of the LEF-Cu method. (**a**) Disinfection efficiency on *E. coli* O157:H7 at various voltages. (**b**) Disinfection efficiency on *P. aeruginosa* PAO1 at various voltages. (**c**) Disinfection efficiency on the virus MS2 at various voltages. Microorganisms are dosed in filtered tap water with a high concentration of 10^6^ CFU/mL (bacteria) or PFU/mL (viruses). The applied voltages range from 0.2 to 3 V and HRT ranges from 0.5 to 3 min. Dashed lines indicate all the microorganisms were inactivated (i.e., live microorganisms were not detected). Error bars represent the standard deviation (*n* = 3).

The disinfection performance of the LEF-Cu system was evaluated using two pathogens (*E. coli* O157:H7 and *P. aeruginosa* PAO1) and one model virus (bacteriophage MS2). As shown in Figure 2a,b, the disinfection efficiency increased with the applied voltage. At voltages below 0.8 V, the inactivation efficiency for the tested bacteria ranged from 0.34- to 1.23-log at 2 min HRT. The variation in performance between the two bacteria may be related to differences in cell structure. Increasing the voltage to 1 V enhanced the inactivation efficiency significantly to 2.7-log, under the same operating condition. When the voltage was further increased to 2 V, pathogens were completely inactivated at 2 min HRT, achieving an inactivation efficiency greater than 6-log (>99.9999% removal). This demonstrated that even low input voltages can generate a high electric field within the device, effectively damaging the microbial cells [28,29,30]. Notably, this voltage level does not lead to significant water decomposition, which is important for minimizing the generation of DBPs, often resulting from chemical reactions [31]. HRT is another crucial factor; longer contact times between the bacterial cells and the electrode leads to higher disinfection efficiencies. When the electric field reaches a lethal threshold for the bacteria, the inactivation rate escalates [32].

Viruses can also be effectively inactivated by the LEF-Cu system, with even higher efficiencies than those observed for bacteria (Figure 2c). At a voltage of 0.5 V and an HRT of 0.5 min, the disinfection performance reached 1.1-log removal. Increasing the voltage to 0.8 V raised the MS2 removal rate to 1.78-log, which is higher than the effect for *E. coli* under the same operating conditions. At a voltage of 1 V, all MS2 were inactivated within 0.5 min, achieving a 6-log removal efficiency (>99.9999% removal), which is 2.77-log higher than that for *E. coli*. This higher efficacy is likely because the functional proteins on viral capsids tend to react with the in-situ released Cu^2+^ easily [25,26].

SEM images show changes in cell structure during disinfection (Figure 2a). Before the LEF-Cu disinfection, the bacteria were intact structures without damage (Figure 2a). After disinfection (1 V; 2 min), pores appeared on the surface of many cells, resulting in the destruction of the cell membrane. Some bacterial cells showed structural disintegration, which likely caused the release of intracellular substances, such as K^+^ [20,25,33]. while also allowing the permeation of external antibacterial agents, such as Cu^2+^. When the voltage was increased to 2 V, the damage intensified. The endoplasmic reticulum, lysosomes, and other cellular structures began to malfunction. For example, lysosomal enzymes may have been released into the cytoplasm, triggering nonspecific degradation of intracellular components and autophagy [34]. Additionally, mitochondrial membranes ruptured, halting ATP production and depleting cellular energy. This led to significant structural damage, increased electrical perforation, and a loss of overall bacterial membrane integrity [35]. These observations suggest that the enhanced electric field destroys the cell membrane, promoting cell death.

**Figure 3:**
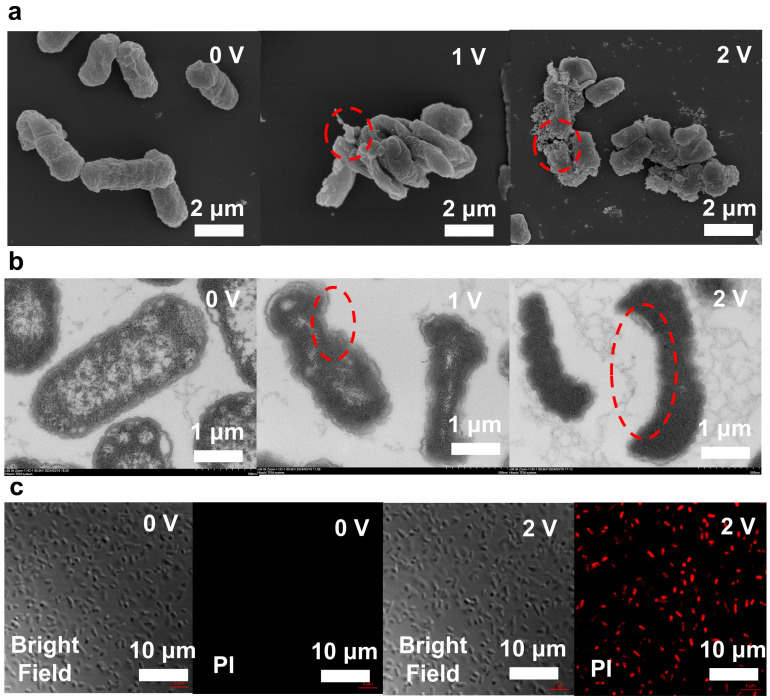
Investigation of disinfection mechanisms. (**a**) SEM images of *E*. *coli* O157:H7 before and after LEF-Cu disinfection. (**b**) TEM images of *E*. *coli* O157:H7 before and after LEF-Cu disinfection. (**c**) Fluorescence confocal images of PI-stained *E*. *coli* O157:H7 before and after LEF-Cu disinfection. Bacteria are dosed in filtered tap water with a high concentration of 10^6^ CFU/mL. The HRT was fixed at 2 min. Red circle indicate the place of cell damage.

The TEM also indicated severe membrane damage of the bacteria after LEF-Cu disinfection (Figure 3b). Compared to untreated *E. coli*, which have intact, smooth, and rounded cell membranes with clearly visible internal structures and regular shapes, the treated *E. coli* (1 V, 2 min) exhibited clear electroporation on the bacterial membrane and the disappearance of internal structures, indicating lethal damage of cell membrane. Under treatment conditions of 2 V for 2 min, the electroporation phenomenon in the cells is more pronounced, with larger pore sizes and cell shrinkage observed, and a loss of their typical shapes, appearing abnormally curved, flattened, or irregular.

The results of the staining test (Figure 3c) also confirmed that *E. coli* cells lost membrane integrity during LEF-Cu treatment. Prior to disinfection, no *E. coli* was stained, indicating that the cell membranes were intact before treatment. However, once a voltage of 2 V was applied, over 90% of the cells were stained, proving that the electroporation reaction occurred in most bacteria, with significant cell damage and an increased percentage of damaged cells. This is consistent with the electroporation mechanism of bacterial inactivation [36].

### 2.2. Destruction of ARGs Using LEF-Cu Method

**Figure 4:**
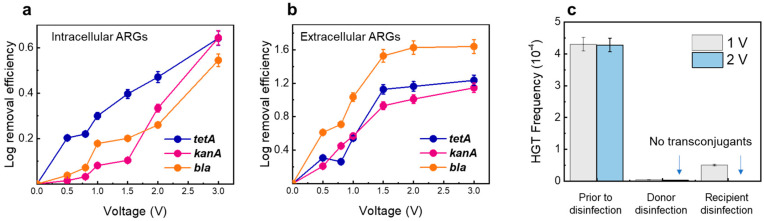
Removal of ARGs using LEF-Cu disinfection. (**a**) Removal performance of iARGs. (**b**) Removal performance of eARGs. (**c**) HGT frequency after LEF-Cu disinfection using *E. coli* (K12) as a donor and *E. coli* (HB101) as a recipient. Bacteria are dosed in filtered tap water with a high concentration of 10^6^ CFU/mL. The applied voltage was set as 2V for pictures (**a**) and (**b**). The HRT was fixed at 2 min for all three pictures. Error bars represent the standard deviation (*n* = 3).

Many disinfection techniques are effective at inactivating organisms but are less effective at destroying their genetic determinants, causing intracellular DNA, such as ARGs to be released after disinfection [10], which may remain in the water and pose a risk of transfer to humans from the One Health perspective. Therefore, the effect of the LEF-Cu method in simultaneously removing ARGs in water was evaluated (Figure 4). During LEF-Cu disinfection, the removal efficiency of ARGs increased significantly with the applied voltage, consistent with the results of microbial inactivation. For example, under conditions of 2 V for 2 min, the removal efficiency of iARGs was 0.54-, 0.64-, and 0.64-log for *tetA*, *kanA*, and *bla*, respectively. The variations in iARG removal may be related to differences in the length, location, and structure of these genes within their host cells.

The LEF-Cu method was also effective at removing eARGs (Figure 4b). At 2 V, the removal of eARGs were 1.09-log (*bla*), 0.59-log (*tetA*), and 0.5-log (*kanA*). This indicates that eARGs have higher opportunities to react with in-situ released Cu^2+^. The effective removal of ARGs is largely due to their hosts being adsorbed onto the surface of the copper anode by static forces, where DNA may undergo despiralization and denaturation under the influence of the local electric field [37,38].

In addition, after LEF-Cu, the ARGs were completely impacted and lost the ability to transfer to new hosts. As shown in Figure 4c, the conjugation transfer frequency, the rate at which the donor cell successfully transfers a plasmid to the recipient cell, of the ARG was 4.28 × 10^−4^. At 1 V only a few transconjugants could be observed. Particularly, after disinfection with an applied voltage of 2 V, the HGT frequency dropped sharply to zero (Figure 4c), potentially because of the effective inactivation of the recipient. In contrast, UV disinfection is inefficient in decreasing the horizontal transfer of ARGs, while chlorination is reported to increase the HGT efficiency [9,39]. Therefore, the LEF-Cu method is efficient in controlling the spread of ARGs in drinking water.

### 2.3. Ions Release and Local Electric Field Contribute to the Microbial Inactivation

**Figure 5:**
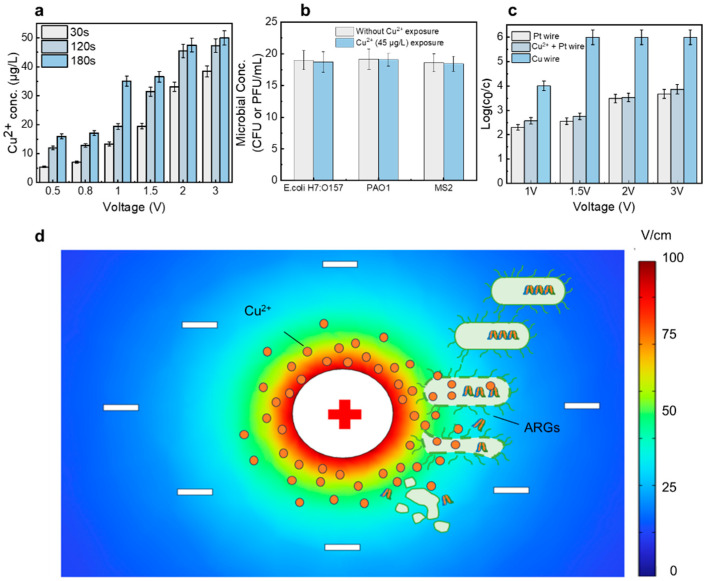
Contribution of Cu^2+^ and local electric field to the LEF-Cu disinfection. (**a**) Cu^2+^ release during LEF-Cu disinfection. (**b**) Toxicity evaluation of the released Cu^2+^. (**c**) Contribution of Cu^2+^ and local electric field to disinfection. (**d**) Electric field simulation and schematic summary of the disinfection device mechanism.

When a voltage of 2 V was applied, 45 μg/L of Cu^2+^ was released into the effluent (Figure 5a). Considering the relatively high drinking water standard of Cu^2+^ (1.3 mg/L for US EPA), the overall toxicity is low. To further confirm the low toxicity of Cu^2+^ to living organisms, live bacteria and viruses were incubated with Cu^2+^ (45 μg/L) for three days. The results showed that all microorganisms remained alive after three days, indicating that Cu^2+^ at inhibitory levels has low toxicity to living organisms and poses limited health risks for drinking (Figure 5b).

Although the in-situ generated Cu^2+^ showed an ineffectiveness to disinfection, it was essential during the LEF-Cu treatment for highly efficient disinfection because the trace Cu^2+^ can significantly improve the antimicrobial performance of the local electric field. To confirm this hypothesis, we used inert platinum (Pt) wire with the same diameter of Cu wire as the anode to investigate the disinfection contribution of the local electric field alone. Under the same operation conditions, the disinfection performance using Pt wire is significantly lower than that using Cu wire (Figure 5c). This is because the in-situ generated Cu^2+^ effectively prompts the inactivation performance of the local electric field. Particularly, once external voltage to the Cu wire is applied, the Cu^2+^ will be significantly enriched near the Cu wire surface, higher than the overall Cu^2+^ in effluent. Since microorganisms commonly carry negative charges in surface water where pH is around neutral, microorganisms will be attracted to the anode (Cu wire) surface. These attracted microorganisms will enter the region with the enhanced electric field and high concentration of Cu^2+^.

The contribution of in-situ generated Cu^2+^ with high localized concentration was further investigated by comparing the disinfection performance using Pt wire and external dosed Cu^2+^ (45 μg/L). Although the water sample containing Cu^2+^ with a concentration similar to the LEF-Cu disinfection and the local electric field was applied, the disinfection efficiency was significantly lower than using the Cu wire anode (Figure 5c). This further indicated the essential contribution of in-situ generated Cu^2+^ near the electrode surface. According to the calculation, the consumption rate of copper wire is 1.1 × 10^−9^ mol/cm^2^/s (Table S4), which supports the consistent use of 18 h before changing. The cost of copper wire is about 0.0045 yuan/m^3^, which is economically affordable. In summary, the LEF-Cu process enables a synergistic disinfection mechanism. The in-situ generated copper ions near the Cu wire surface can be effectively permeated into the electroporated microorganisms caused by the local electric field to damage basic groups of genes through complex reactions (Figure 5d).

### 2.4. Practical Application of LEF-Cu Method

**Figure 6:**
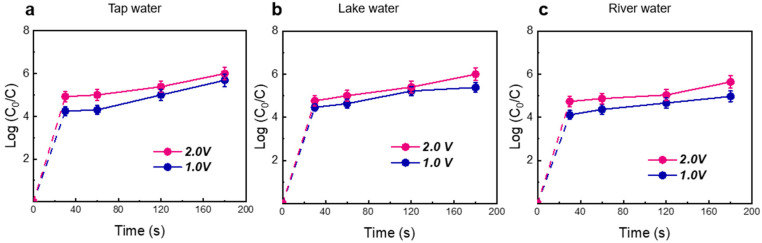
Disinfection efficacy of LEF-Cu method for treating tap water (**a**), lake water (**b**), and river water (**c**). *E. coli* H7:O157 was dosed in filtered water samples at 10^6^ CFU/mL. Error bars represent the standard deviation (*n* = 3).

To examine the flexibility of the LEF-Cu method, water samples in actual conditions (including tap water, lake water, and river water) were dosed with pathogens (*E. coli* OH157:H7). As shown in Figure 6a–c, when applied with 2.0 V voltage and 2 min HRT, LEF-Cu achieved complete disinfection (6-log) in all tested water (>99.9999% removal). This indicated that the water matrix of the surface showed minimal impact on disinfection. Furthermore, the LEF-Cu disinfection effectively reduced the HGT frequency of ARGs. After disinfection with 2.0 V voltage and 2 min HRT, the conjugative transfer frequencies of resistance genes in all three environmental samples dropped to zero (Figure S1), highlighting significant efficacy in controlling the risk of transmitting antibiotic resistance.

Owing to the specific disinfection mechanism of Cu^2+^-assisted physics-based electroporation, only 2 V of voltage is required for complete disinfection of harmful microorganisms and genes in river water at high throughput (HRT of 2 min). The energy consumption (10^−2^ kWh/m^3^) of the LEF-Cu method is significantly lower than conventional UV disinfection (~10^2^ kWh/m^3^). LEF-Cu disinfection has great potential for decentralized applications, especially in regions with insufficient sanitation and power supply. Thus, the LEF-Cu method can readily provide safe drinking water to protect people from pathogens and related diseases caused by harmful genes and directly improve public health in these low-resource countries.

## 3. Materials and Methods

### 3.1. Materials

*E. coli* O157:H7 (NCTC12900) was acquired from Huankai Microbial Technology Co., Ltd., (Guanzhou, China). *E. coli* K12 (carrying RP4 plasmid with ARGs of *tetA*, *bla*, and *kanA* genes), *E. coli* HB101, and *Pseudomonas aeruginosa* (*P. aeruginosa*) PAO1 (CGMCC 1.12483) were obtained from the China Center of Industrial Culture Collection. These bacterial strains were preserved by low-temperature glycerol storage and kept in a −80 °C freezer. *Escherichia coli* bacteriophage *E. coli* MS2 (ATCC 15597-B1), used as the model virus in our study, was obtained from the American Type Culture Collection. Copper wire and copper sheet was purchased from Tengfeng Metal Material Co., Ltd. (Handan, China).

### 3.2. Construction of the Disinfection Device

**Figure 7:**
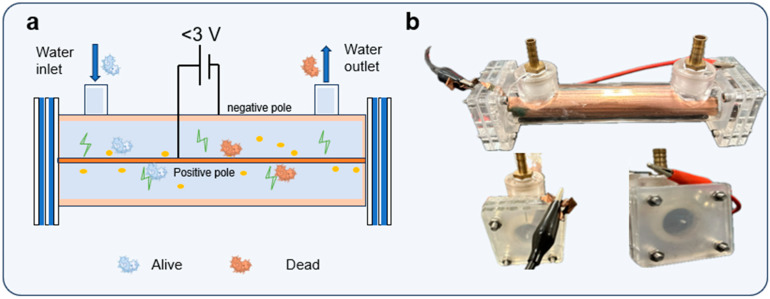
(**a**) Schematic diagram showing the LEF-Cu disinfection device. (**b**) View of the LEF-Cu disinfection device.

The LEF-Cu disinfection device comprises a chamber and a support frame constructed from plexiglass to simulate the household water pipe. It features two brass pipe connectors positioned on opposite sides, serving as the inlet and outlet respectively (Figure 7a). The inner diameter and length of the tube were 2 cm and 15 cm, respectively, having an effective volume of 47.1 mL. A copper wire, with 100 μm in diameter, was installed centrally across the reactor and functions as the anode. Aligned with the central axis, a copper sheet firmly attached to the tube wall acted as the cathode. When external voltage was applied, this configuration generated an intensified non-uniform electric field around the central electrode [40]. Before operation, the copper wire was cleaned with dilute hydrochloric acid (0.1 M) to remove any surface oxide layer. A programmable peristaltic pump (Longer L100-1E, Shanghai, China) facilitated the continuous flow of water into the device through the inlet.

### 3.3. Disinfection Experiment

*E. coli* O157:H7, *P. aeruginosa* PAO1, and *E. coli* K12 were cultured at 37 °C for 12 h and harvested by centrifugation at 5000 rpm. The cultured pathogens were washed with sterile deionized water and diluted to a bacterial concentration of 10^9^ colony-forming units per mL (CFU/mL). Each type of pathogen was then disinfected independently through the LEF-Cu disinfection device. The MS2 bacteriophage stock solution, initially at a concentration of 10^13^ plaque-forming units per mL (PFU/mL), was diluted with DI water to achieve a final concentration of 10^9^ PFU/mL. These prepared water samples, containing either pathogens or MS2, were dosed into tap water with a final concentration of 10^6^ CFU/mL or PFU/mL and then were pumped into the LEF-Cu device with a flow rate of 94.2–15.7 mL/min, detected by the programmable peristaltic pump (L100-1E, Longer Pump), corresponding to hydraulic retention times (HRTs) from 30 to 180 s. A voltage of 0.2 to 3 V was applied to the electrodes by a potentiostat (CHI600E, CH Instruments, Austin, TX, USA). Water samples were collected from the outlet before and after disinfection for further pathogen and gene quantification. To investigate the impact of different electrode materials on the disinfection efficacy, the copper wire and copper sheet were substituted with a pure platinum wire of identical dimensions (100 μm diameter) and platinum sheet for the same disinfection operation conditions. For the disinfection experiment at each dose, three parallel samples were processed.

For bacterial samples, the effluent before and after disinfection was serially diluted by a factor of 10, ranging from 10^−1^ to 10^−7^. To perform each dilution, 100 μL of the previous dilution was added to 900 μL of 0.1 M PBS, followed by thorough mixing. Each dilution (100 μL) was spread on the Luria–Bertani (LB) agar. Colony counts were performed on plates containing fewer than 200 colonies to ensure accuracy. The MS2 was mixed with the *E. coli* host mixture and inoculated onto LB agar, followed by incubation at 37 °C. After 12 h, the plaques from samples before and after disinfection were counted. Each sample at each dilution was plated in triplicate. The microbial removal efficiency (*E*) was calculated using the following Equation (1) [41]:

(1) $$E=log\frac{C_{0}}{C}$$

where *C*_0_ represents the initial bacterial and viral concentration before disinfection, and *C* represents the concentration of alive bacteria and viruses after disinfection.

### 3.4. ARG Quantification

To ensure accuracy and consistency, each qPCR experiment included three technical replicates. The *E. coli* K12-containing RP4 plasmid before and after disinfection was filtered using a 0.22 μm sterile membrane (Jin Teng Tech, Tianjin, China). The membrane was collected for intracellular DNA (iDNA) extraction, and the filtered water was used to extract extracellular DNA (eDNA). For iDNA extraction, the filter membrane was cut into 0.3 cm × 0.3 cm pieces and vortex (Kylin-Bell Company, Haimen, Nantong, China) mixed with 3 mL SLX-Mlus buffer containing 500 mg Glass Beads X to facilitate DNA transfer from the membrane to the solution. The iDNA was extracted using the Water DNA Kit (OMEGA BIO-TEK, Norcross, GA, USA) following the manufacturer’s instructions. All extracted DNA was quantified by Nanodrop (IMPLEN company, Munich, Germany) to ensure the quality of DNA.

For the extraction of eDNA, the magnetic bead method was employed based on our previous research [42]. Specifically, 5 mL of the filtrate was combined with 4 mL of Buffer CL (Biomagbeads, Wuxi, China) and 3 mL of isopropyl alcohol in a 50 mL centrifuge tube. After adding 30 μL of magnetic beads, the mixture was thoroughly mixed by vortices (Kylin-Bell Company, Haimen, Nantong, China) and the magnetic beads were adsorbed with magnets to complete the separation from the mixed liquid. The magnetic beads, with the adsorbed DNA, were washed three times using 1 mL of Buffer CW1 (Biomagbeads, Wuxi, China) and 1 mL of Buffer CW2 (75% ethanol). The DNA was then eluted from the magnet using 30 μL of pre-heated elution buffer at 55 °C.

Three ARGs (*tetA, bla* and *kanA*) were quantified using real-time quantitative polymerase chain reaction (qPCR) (BIO-Rad, Hercules, CA, USA), with all primers validated prior to use (Table S1). The qPCR experiments were conducted in eight-strip tubes, with each reaction having a total volume of 20 μL, consisting of 6.4 μL of DI water, 2 μL of template DNA, 100 μM forward primer and reverse primer 0.8 μL each (Sangon Biotech, Shanghai, China), and 10 μL of MIX enzyme SYBR Green I (TSINGKE Company, Beijing, China), in which MIX enzymes act as the main mixture. The qPCR was performed using a CFX Connect real-time fluorescence PCR instrument (BIO-Rad), under the following conditions: 40 cycles starting with a 15-min preheating at 95 °C to activate the reaction mixture, followed by denaturation at 95 °C for 10 s, a 20-s annealing step, and a melt curve analysis to assess product specificity. To ensure accuracy and consistency, each qPCR experiment included three technical replicates, and the standard curves of the above three genes construction followed the same protocol.

### 3.5. Bacterial Morphology Characterization

Scanning electron microscopy (SEM) was used for the bacterial morphology characterization. The water samples containing bacteria before and after disinfection were centrifuged and then the harvested cells were fixed with 2.5% glutaraldehyde solution at 4 °C for 12 h. Following fixation, the samples were rinsed three times using 0.1 M PBS, and then dehydrated in a series of ethanol solutions with increasing concentrations (50%, 70%, 80%, 90%, and 100%). Subsequently, the ethanol was replaced with 100% tert-butanol. After freeze-drying and gold sputtering, the morphology of the bacterial cell surface was examined using an SEM (Hitachi, Regulus-8100, Tokyo, Japan) [43]. A transmission electron microscope (TEM) was also used to characterize the bacterial structure. The harvested bacterial samples were rinsed three times with PBS buffer before being fixed in 1% osmium acid for 1 h, followed by three rinses in 0.1 M PBS. The samples were then dehydrated through a graded series of ethanol solutions (30%, 50%, 70%, 90%, and 100%), followed by two applications of 100% propanol. The samples were then infiltrated, embedded, and polymerized [44]. The treated bacteria were analyzed using TEM (Hitachi, HT7800, Tokyo, Japan) to assess structural changes in the bacterial cells before and after disinfection.

To further examine structural changes following disinfection, a laser scanning confocal microscope (Carl Zeiss, LSM 900, Oberkochen, Germany) was utilized [45]. Bacteria before and after disinfection were collected, adjusted to a concentration of approximately 10^5^ cells/mL, and re-suspended using the dye solution C in the kit (Bestbio, Shanghai, China). After gentle mixing, 20 μM propidium iodide (PI) was then added to the bacteria for incubation in the dark at 4 °C for 30 min. Laser scanning microscopy (LSM) images with red fluorescence (535 nm of excitation) were then captured.

### 3.6. Conjugation Experiment

To examine the effect of LEF-Cu on the potential of horizontal gene transfer (HGT) of ARGs, the conjugation experiment was conducted by using *E. coli* K12 carrying the RP4 plasmid as the donor, while *E. coli* HB101 as the recipient. Five mL samples containing either the donor or recipient were processed for disinfection under 1 V and 2 V applied voltage and 2 min HRT. Following disinfection, the samples were cultured at 200 rpm in an incubator at 37 °C with vortex oscillation for 8 h. The 100 μL cultured mixture was then diluted 10–10^7^ times and screened on LB Agar plates containing different antibiotics. Transconjugants were screened using ampicillin, kanamycin, and streptomycin at a concentration of 20 μg/mL, while streptomycin at 20 μg/mL was used to screen the recipients. The presence of ARGs in the transconjugants was confirmed by PCR testing. The total reaction system was 20 μL, including sterile water 6.4 μL, template DNA 2 μL, forward and reverse primer 0.8 μL (kanA, with the sequence shown in Table S1), and MIX enzyme 10 μL. The entire amplification process consists of 40 cycles, starting with a 15 min preheating treatment at 95 °C. The entire amplification process starts with a 15 min preheating treatment at 95 °C, followed by 35 cycles of 95 °C for 10 s, 55 °C for 30 s, and 72 °C for 30 s.

### 3.7. Determination of the Effect of Cu^2+^

The release of Cu^2+^ during disinfection was examined. One mL of the effluent samples after disinfection was filtrated and the filtrate was digested with 10 mL of pure HNO_3_ overnight and then was evaporated at 95 °C. HNO_3_ (2%) was used to resuspend the sample, and then the ions concentration was analyzed using ICP-OES (PerkinElmer, Norfolk County, MA, USA).

To investigate the disinfection mechanism of the device, the effects of Cu^2+^ disinfection were examined. An *E. coli* O157:H7 suspension was precisely adjusted to 10^6^ CFU/mL and spiked with Cu^2+^ at a set concentration. The mixed solution was processed for disinfection by the local electric field device, with a pure platinum wire (100 μm diameter) as the anode, under the same disinfection conditions. Samples were taken from the treated solution and evaluated using the dilution plating method to assess the disinfection effect under the same copper ion concentration conditions.

### 3.8. Performance Investigation in Actual Water Media

To evaluate the efficacy of the disinfection device in different waterbodies, samples of tap water (collected from Nanjing Water Group drinking water treatment plant), lake water (collected from Yangshan Lake in Nanjing Province), and Yangtze River water were collected from Nanjing, China; five L of each water sample was collected. Detailed water quality parameters are provided in Table S2. Following filtration and sterilization, each sample was inoculated with 10^6^ CFU/mL of *E. coli* O157:H7. The contaminated samples were then processed through the disinfection device at voltages of 1 V and 2 V, respectively, with a reaction time of 120 s for each. After treatment, the effluent samples were collected, serially diluted, and cultured using the spread plate method (LB media) to assess the disinfection performance by counting the remaining viable bacteria.

### 3.9. COMSOL Simulation

To elucidate the mechanism of LEF-Cu, the electric field distribution within the device was simulated using finite element analysis. The COMSOL Multiphysics software 6.0 was employed for this purpose (additional information is available in Table S3). A geometric model was constructed based on parameters such as the distance between electrodes, the dimensions of the positive and negative electrodes in the reaction chamber, and boundary conditions. The physical field was set under a voltage of 2 V, and finite element meshing was performed to simulate and visualize the electric field distribution. The electric field Equations (2) and (3) are as follows:

(2) E=−∇V

(3) $$D=\varepsilon_{0}\varepsilon_{r}E$$

*E*: Electric Field Intensity

V: Electric Potential

D: Electric Displacement Vector

### 3.10. Data Analysis

Statistical analysis was performed using Origin 2018 (Origin Lab Corporation, Northampton, MA, USA) and SPSS 19.0 (IBM, Armonk, NY, USA). Student *t*-test was applied to examine the significant difference between the two results at a *p*-value of 0.05 [46].

## 4. Conclusions

In summary, we developed a LEF-Cu disinfection method that can simultaneously inactivate pathogens and ARGs in drinking water using LEF-Cu without external chemical dosing. A thin copper wire placed in the center of a water pipe can generate LEF and trace copper ions after an external low voltage is applied. This setup successfully achieved complete inactivation of pathogens and viruses with 2 V external voltage under low-energy consumption (10^−2^ kWh/m^3^) and can effectively disrupt ARGs for inhibiting the spread of harmful genes. Adaptable to various water conditions, this disinfection device holds considerable potential for mitigating the transmission of pathogens and their related high-risk genes and can provide reliable safe drinking water for household applications.

While the LEF-Cu device shows great potential for pathogen inactivation and ARG removal, practical application and scaling up present challenges that will require further research. First, the device configuration requires optimization for high-volume treatment, including optimizing the types of metal wire, such as silver, as well as determining the optimal electric anode length, HRT, and applied voltage. Second, the stability and potential degradation of the copper wire must be thoroughly investigated. To eliminate potential risks of copper to human and ecosystem health, future research should focus on minimizing copper ion release, such as by employing nanowire materials loaded on the central electrode in low-voltage mode. A more in-depth study of the inactivation mechanisms should be conducted to explore effects beyond the LEF and ions, such as the potential generation of free radicals, even though this is unlikely under the conditions used in this study. Continuous operation and development are essential for refining this method and enhancing its potential to protect human health.

## Data Availability

Data are contained within the article and Supplementary Materials.

## Supplementary Materials

The following supporting information can be downloaded at: https://www.mdpi.com/article/10.3390/antibiotics13121161/s1, Table S1: Primers used in this study; Table S2: Quality of water samples collected in this study; Table S3: Parameters used for the simulations of electric field, and *E. coli* distribution in CECIC; Table S4. Copper flux calculation from electrochemical releasing; Figure S1. Disinfection performance of LEF-Cu method on the horizontal gene transfer frequency for treating tap water.
