# Supplementary material for: Local Electric Field-Incorporated In-Situ Copper Ions Eliminating Pathogens and Antibiotic Resistance Genes in Drinking Water
Source: Antibiotics (Basel). 2024 Dec 2;13(12):1161. doi: 10.3390/antibiotics13121161 (PMC11672500; doi:10.3390/antibiotics13121161)
Supplement: Supplementary file 1 [file antibiotics-13-01161-s001.zip › antibiotics-3298309-supplementary.pdf]

Supplementary Information (SI)

Local electric field-incorporated in-situ copper ions eliminating pathogens and antibiotic resistance genes in drinking water

Ruiqing Li[1], Haojie Dai[2], Wei Wang[1], Rulin Peng[1], Shenbo Yu[3], Xueying Zhang[1], Zheng-Yang Huo[4]*, Qingbin Yuan[1,3]*, Yi Luo[3]

[1] School of Environmental Science and Engineering, Nanjing Tech University, Nanjing 211816, China

[2] State Key Laboratory of Pollution Control and Resource Reuse, School of the Environment, Nanjing University, Nanjing 210023, China

[3] School of Environment and Natural Resources, Renmin University of China, Beijing 100872, China

[4] School of Chemistry and Life Resources, Renmin University of China, Beijing 100872, China

Corresponding authors: zhengyanghuo.edu@ruc.edu.cn (Z.-Y.H.); yuanqb@nju.edu.cn (Q.Y.)

Table S1. Primers used in this study.

DNA	Forward primer (5’-3’)	Reverse primer (5’-3’)	Amplification length（bp）
tetA	CGGTCTTCTTCATCATGCAAC	GTCCCAGTGAAAGCGATCC	79
Bla	CTACGATACGGGAGGGCTTA	ATAAATCTGGAGCCGGTAGA	81
KanA	TGAAATAGGCTATCTGCCGA	GTGATCTCCGAACATTTCTC	139

Table S2. Quality of water samples collected in this study.

Sample	Location (N, E)	NH_3- N (mg/L)	COD (mg/L)	Turbidity (NTU)	Total phosphorus (mg/L)	TDS (mg/L)	pH
Tap water	32° 11’N<br>118° 95’E	0.13	11.7	0.35	0.05	209	7.65
Lake water	32° 10’N<br>118° 94’E	0.21	7.1	23.50	0.10	325	7.51
River water	31° 96’N<br>118° 82’E	0.24	14.8	46.77	0.12	386	7.63

Table S3. Parameters used for the simulations of electric field, and *E. coli* distribution in CECIC.

Parameter	Unit	Value
Radius of the center electrode (R)	cm	1
Radius of the center electrode (r)	cm	0.005
Electrical conductivity of medium	S/m	5.5×10^{-6}
Relative Permittivity of medium (εf)	1	81
Vacuum permittivity ($\varepsilon 0$)	F/m	8.85×10^{-12}
Electric potential (V)	V	3
Positive charge number of copper ion (zi)	1	2
Relative permittivity of a particle (εp)	1	100
Positive charge number of a particle	1	-10^{6}

Table S4. Copper flux calculation from electrochemical releasing

Parameter	Unit	Value	Note
Radius of the outer electrode (R)	cm	1	Reactor size
Radius of the center electrode (r)	cm	0.005	Reactor size
Length of LEF-Cu (L)	cm	15	Reactor size
Total volume of the LEF-Cu (V)	mL	47.1	V=$L \times \pi \times R^2$
Surface area of center electrode (A)	cm^2	0.47	A=$\pi \times d \times L$
Average operation current (I)	μA	100	Measured during experiment
Number of charges per particle (z)	#	2	For copper ions
Faraday constant	C/mol	96485	
Cu release flux (v)	$mol/cm^2/s$	$1.1*10^{-9}$	v=$\frac{I}{z \times F \times A}$
Copper wire price	$yuan/m^3$	0.0045	

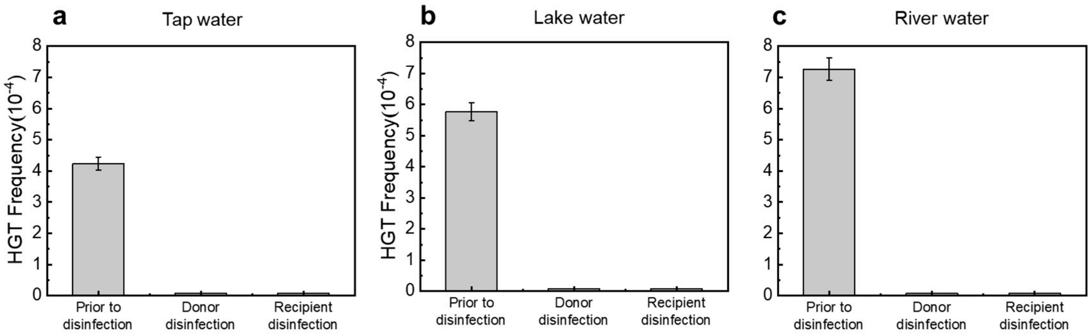

Figure S1. Disinfection performance of LEF-Cu method on the horizontal gene transfer frequency for treating tap water (a), lake water (b), and river water (c). The HRT was fixed at 2 min. Error bars represent the standard deviation (n = 3).
